# Supplementary material for: Sleep in the Natural Environment: A Pilot Study
Source: Sensors (Basel). 2020 Mar 3;20(5):1378. doi: 10.3390/s20051378 (PMC7085707; doi:10.3390/s20051378)
Supplement: Supplementary file 1 [file sensors-20-01378-s001.pdf]

sensors

MDPI

Supplementary Materials

Sleep in the Natural Environment: A Pilot Study

Fayzan F. Chaudhry[1,2+], Matteo Danieletto[1,2,3+], Eddye Golden[1,2,3], Jerome Scelza[2,3], Greg Botwin[2,3], Mark Shervey[2,3], Jessica K. De Freitas[1,2,3], Ishan Paranjpe[1], Girish N. Nadkarni[1,4,5], Riccardo Miotto[1,2,3], Patricia Glowe[1,2,3], Greg Stock[3], Bethany Percha[2,3], Noah Zimmerman[2,3], Joel T. Dudley[2,3*], Benjamin S. Glicksberg[1,2,3*]

[1] Hasso Plattner Institute for Digital Health at Mount Sinai, Icahn School of Medicine at Mount Sinai, New York, NY, 10032, USA.
[2] Department of Genetics and Genomic Sciences, Icahn School of Medicine at Mount Sinai, New York, NY, 10032, USA
[3] Institute for Next Generation Healthcare, Icahn School of Medicine at Mount Sinai, New York, NY, 10032, USA.
[4] The Charles Bronfman Institute for Personalized Medicine, Icahn School of Medicine at Mount Sinai, New York, New York.
[5] Department of Medicine, Icahn School of Medicine at Mount Sinai, New York, New York.

+ Authors contributed equally
* Correspondence: joel.dudley@mssm.edu or benjamin.glicksberg@mssm.edu

S1: Demographic questionnaire

Demographics

1. Assigned Study Identification Number:

2. What is your biological sex?

- Male
- Female

3. Are you Spanish, Hispanic, or Latino?

- No
- Yes

4. What is your race? (Mark all that apply)

- White
- Black/African American
- American Indian/Alaska Native
- Asian
- Native Hawaiian or Other Pacific Islander
- Other (please specify)

5. What is the highest level of education you have completed?

6. What is your height in inches?

7. What is your weight in lbs?

8. What is your neck collar size in cms?

9. What best describes your current main daily activities?

- Working full time (day shifts)
- Working full time (rotating or night shifts)
- Working part time (day shifts)
- Working part time (rotating or night shifts)
- Unemployed, looking for work
- In school
- Stay at home parent
- Retired
- Disabled
- Prefer not to answer

10. Thinking about the past 3 months, which of the following best describes your work schedule

- Regular day shifts
- Regular evening shifts
- Regular night shifts (9pm to 8am)
- Rotating shifts (one that changes periodically from days, evening or nights)
- Split shifts (one consisting of two distinct periods each day)
- An irregular schedule
- Prefer not to answer

11. Do you have flexible work hours that allow you to vary or make changes in the time you begin and end work?

- Yes
- No
- Prefer not to answer

S2: SF-36 questionnaire

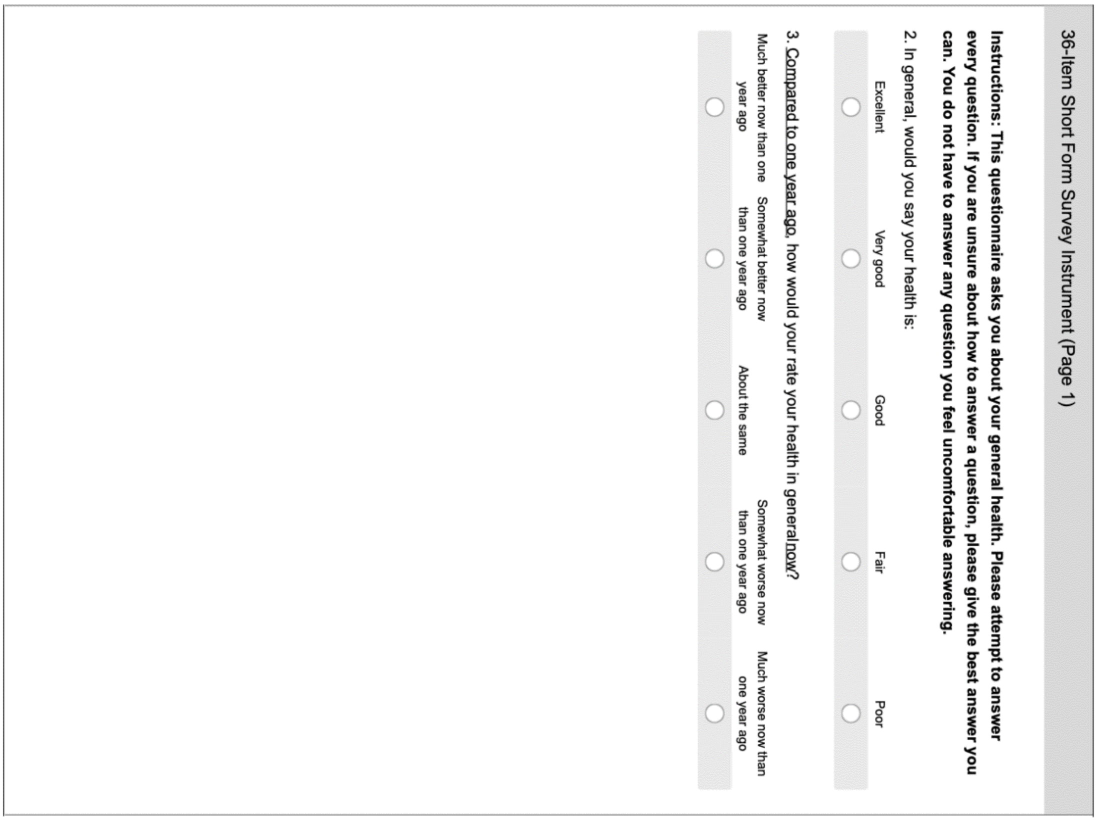

36-Item Short Form Survey Instrument (Page 1)

Instructions: This questionnaire asks you about your general health. Please attempt to answer every question. If you are unsure about how to answer a question, please give the best answer you can. You do not have to answer any question you feel uncomfortable answering.

2. In general, would you say your health is:

Excellent ◯ Very good ◯ Good ◯ Fair ◯ Poor ◯

3. Compared to one year ago, how would your rate your health in generalnow?

Much better now than one year ago ◯ Somewhat better now than one year ago ◯ About the same ◯ Somewhat worse now than one year ago ◯ Much worse now than one year ago ◯

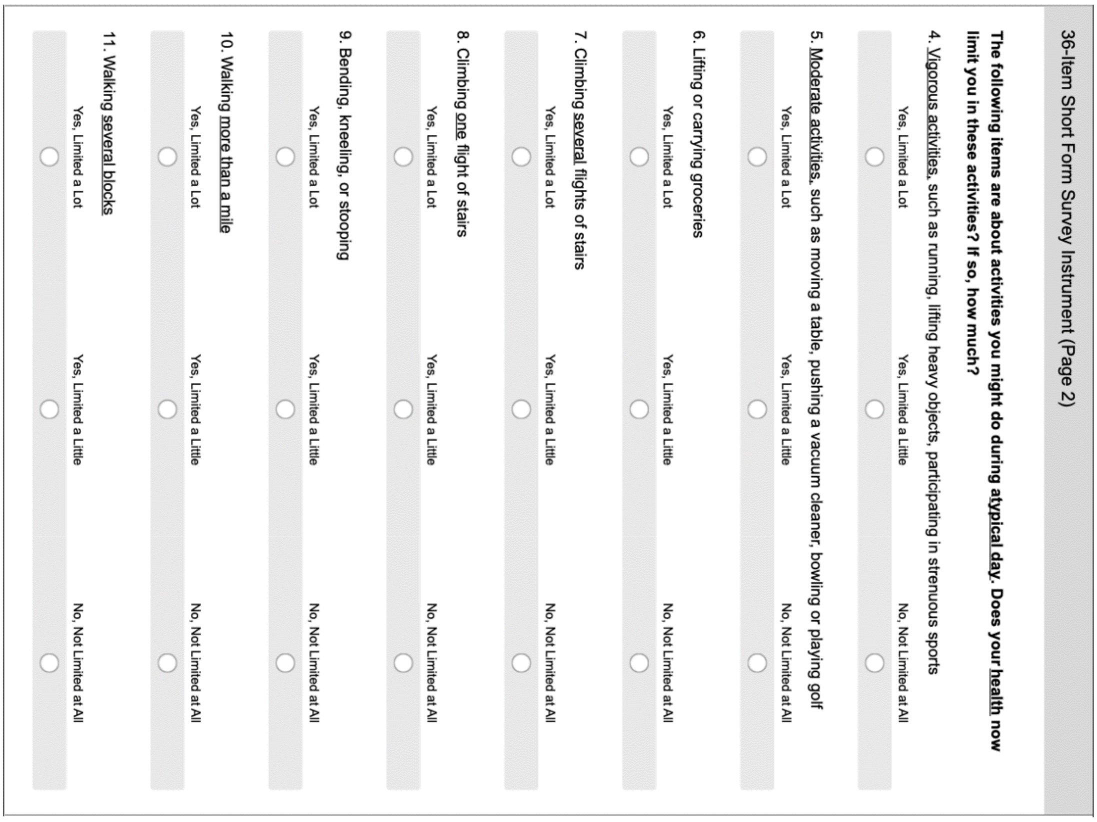

36-Item Short Form Survey Instrument (Page 2)

The following items are about activities you might do during atypical day. Does your health now limit you in these activities? If so, how much?

4. Vigorous activities, such as running, lifting heavy objects, participating in strenuous sports

Yes, Limited a Lot ◯ Yes, Limited a Little ◯ No, Not Limited at All ◯

5. Moderate activities, such as moving a table, pushing a vacuum cleaner, bowling or playing golf

Yes, Limited a Lot ◯ Yes, Limited a Little ◯ No, Not Limited at All ◯

6. Lifting or carrying groceries

Yes, Limited a Lot ◯ Yes, Limited a Little ◯ No, Not Limited at All ◯

7. Climbing several flights of stairs

Yes, Limited a Lot ◯ Yes, Limited a Little ◯ No, Not Limited at All ◯

8. Climbing one flight of stairs

Yes, Limited a Lot ◯ Yes, Limited a Little ◯ No, Not Limited at All ◯

9. Bending, kneeling, or stooping

Yes, Limited a Lot ◯ Yes, Limited a Little ◯ No, Not Limited at All ◯

10. Walking more than a mile

Yes, Limited a Lot ◯ Yes, Limited a Little ◯ No, Not Limited at All ◯

11. Walking several blocks

Yes, Limited a Lot ◯ Yes, Limited a Little ◯ No, Not Limited at All ◯

12. Walking one block

Yes, Limited a Lot	Yes, Limited a Little	No, Not Limited at All
○	○	○

13. Bathing or dressing yourself

Yes, Limited a Lot	Yes, Limited a Little	No, Not Limited at All
○	○	○

Medical Outcomes Study: 36-Item Short Form Survey Instrument (Page 3)

During the past 4 weeks, have you had any of the following problems with your work or other regular daily activities as a result of your physical health?

14. Cut down the amount of time you spent on work or other activities

Yes	No
○	○

15. Accomplished less than you would like

Yes	No
○	○

16. Were limited in the kind of work or other activities

Yes	No
○	○

17. Had difficulty performing the work or other activities (for example, it took extra effort)

Yes	No
○	○

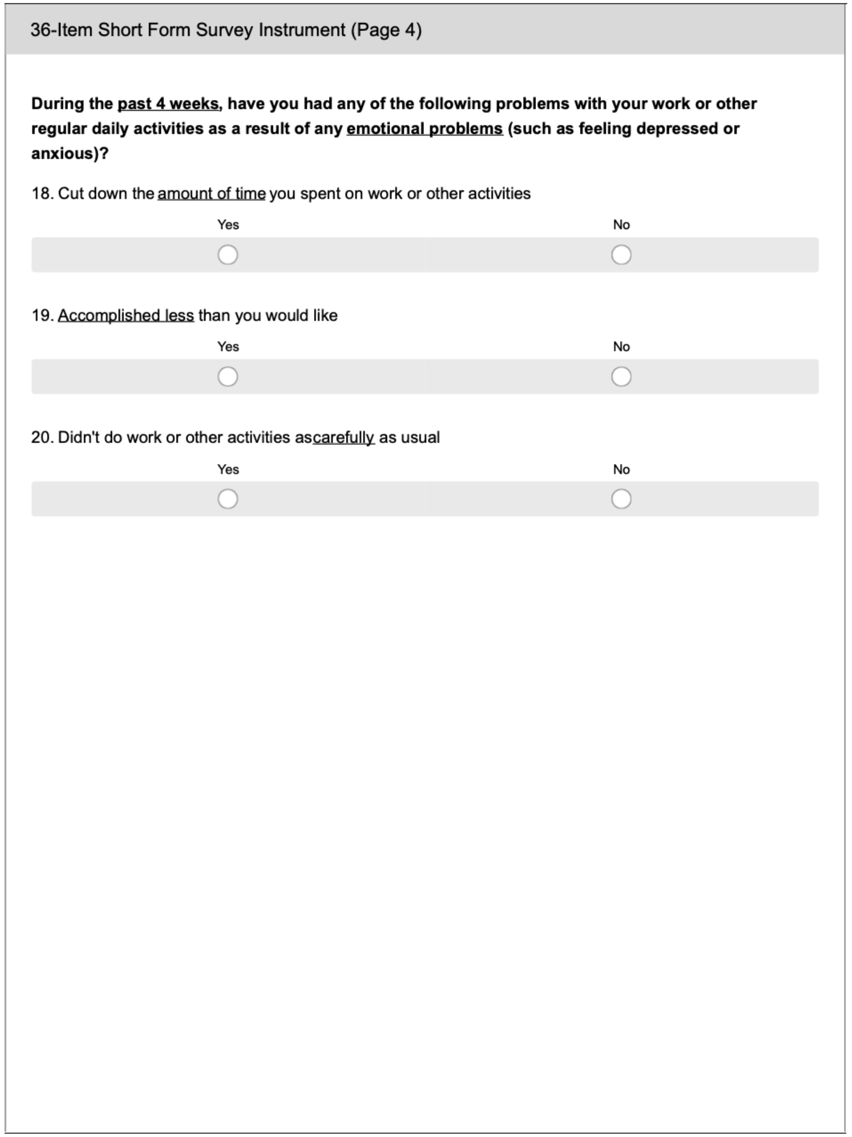

36-Item Short Form Survey Instrument (Page 4)

During the past 4 weeks, have you had any of the following problems with your work or other regular daily activities as a result of any emotional problems (such as feeling depressed or anxious)?

18. Cut down the amount of time you spent on work or other activities

Yes	No
○	○

19. Accomplished less than you would like

Yes	No
○	○

20. Didn't do work or other activities as carefully as usual

Yes	No
○	○

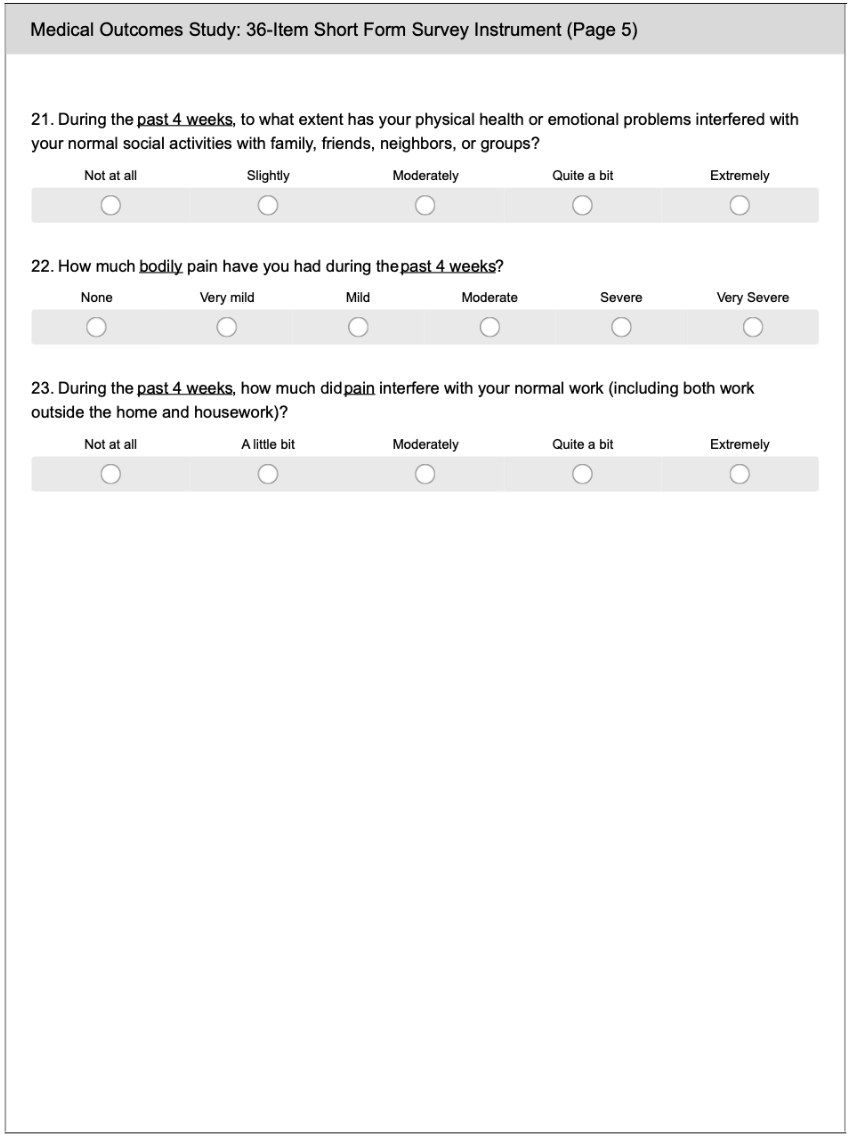

Medical Outcomes Study: 36-Item Short Form Survey Instrument (Page 5)

21. During the past 4 weeks, to what extent has your physical health or emotional problems interfered with your normal social activities with family, friends, neighbors, or groups?

Not at all	Slightly	Moderately	Quite a bit	Extremely
○	○	○	○	○

22. How much bodily pain have you had during the past 4 weeks?

None	Very mild	Mild	Moderate	Severe	Very Severe
○	○	○	○	○	○

23. During the past 4 weeks, how much did pain interfere with your normal work (including both work outside the home and housework)?

Not at all	A little bit	Moderately	Quite a bit	Extremely
○	○	○	○	○

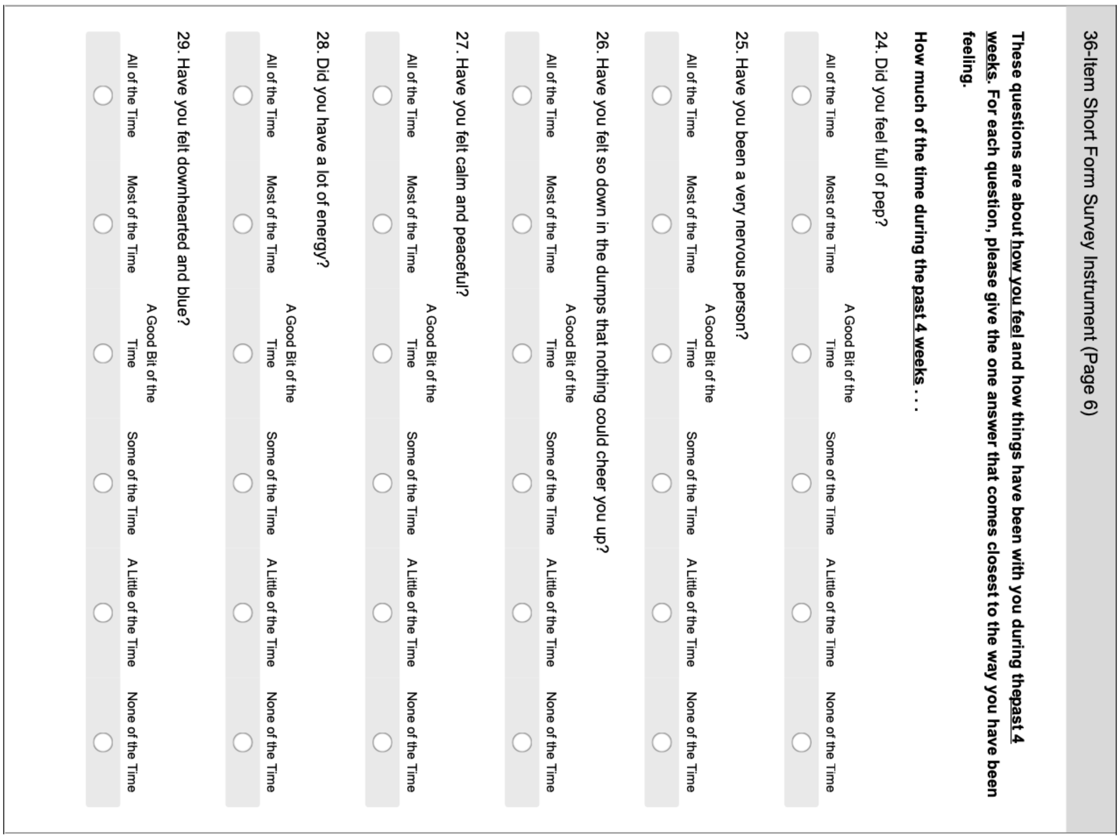

36-Item Short Form Survey Instrument (Page 6)

These questions are about how you feel and how things have been with you during thepast 4 weeks. For each question, please give the one answer that comes closest to the way you have been feeling.

How much of the time during the past 4 weeks . . .

Question	All of the Time	Most of the Time	A Good Bit of the Time	Some of the Time	A Little of the Time	None of the Time
24. Did you feel full of pep?	○	○	○	○	○	○
25. Have you been a very nervous person?	○	○	○	○	○	○
26. Have you felt so down in the dumps that nothing could cheer you up?	○	○	○	○	○	○
27. Have you felt calm and peaceful?	○	○	○	○	○	○
28. Did you have a lot of energy?	○	○	○	○	○	○
29. Have you felt downhearted and blue?	○	○	○	○	○	○

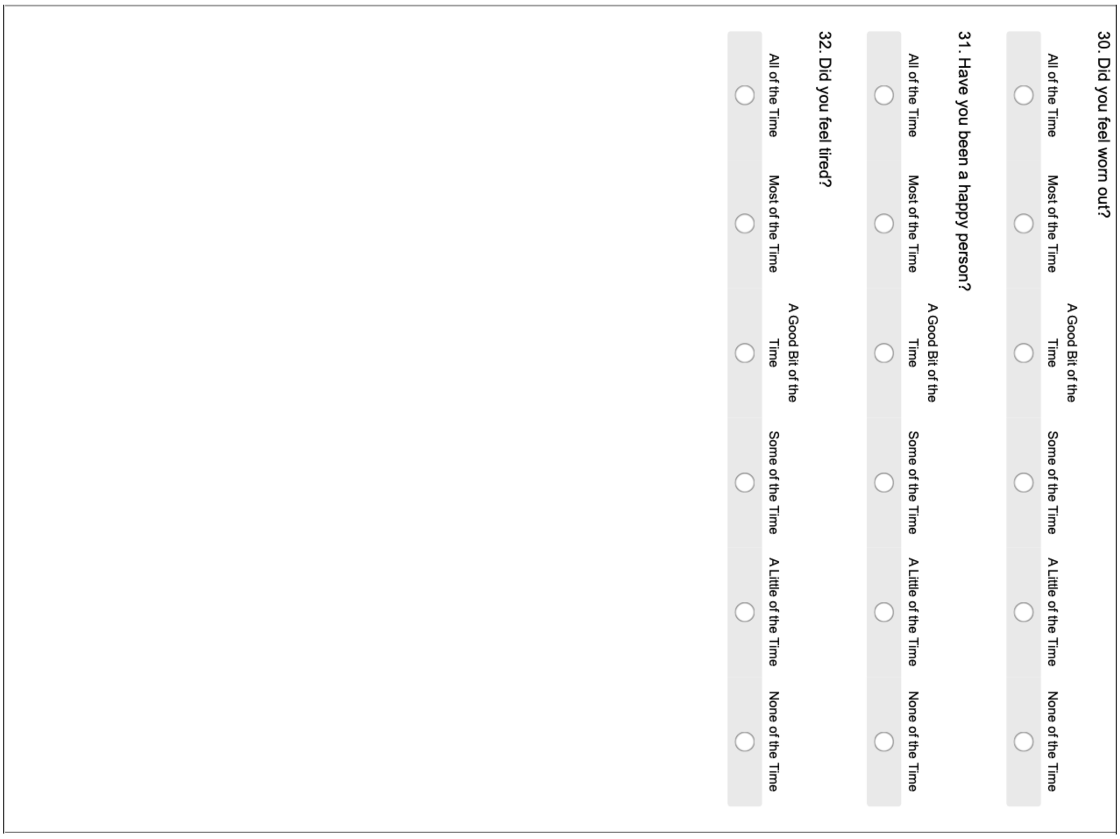

Question	All of the Time	Most of the Time	A Good Bit of the Time	Some of the Time	A Little of the Time	None of the Time
30. Did you feel worn out?	○	○	○	○	○	○
31. Have you been a happy person?	○	○	○	○	○	○
32. Did you feel tired?	○	○	○	○	○	○

36-Item Short Form Survey Instrument (Page 7)

33. During the past 4 weeks, how much of the time has yourphysical health or emotional problems interfered with your social activities (like visiting with friends, relatives, etc.)?

All of the time	Most of the time	Some of the time	A little of the time	None of the time
○	○	○	○	○

36-Item Short Form Survey Instrument (Page 8)

How TRUE or FALSE is each of the following statements for you.

34. I seem to get sick a little easier than other people

Definitely True	Mostly True	Don't Know	Mostly False	Definitely False
○	○	○	○	○

35. I am as healthy as anybody I know

Definitely True	Mostly True	Don't Know	Mostly False	Definitely False
○	○	○	○	○

36. I expect my health to get worse

Definitely True	Mostly True	Don't Know	Mostly False	Definitely False
○	○	○	○	○

37. My health is excellent

Definitely True	Mostly True	Don't Know	Mostly False	Definitely False
○	○	○	○	○

S3: MEQ questionnaire

MORNINGNESS-EVENINGNESS QUESTIONNAIRE (MEQ)

Instructions: This questionnaire asks you about when you feel the most alert. Please attempt to answer every question. If you are unsure about how to answer a question, please give the best answer you can. You do not have to answer any question you feel uncomfortable answering. Each question should be answered independently of others. Do NOT go back and check your answers.

38. Approximately what time would you get up if you were entirely free to plan your day?

5:00 AM–6:30 AM (05:00–06:30 h)	6:30 AM–7:45 AM (06:30–07:45 h)	7:45 AM–9:45 AM (07:45–09:45 h)	9:45 AM–11:00 AM (09:45–11:00 h)	11:00 AM–12 noon (11:00–12:00 h)	12 noon-5:00 AM (12:00-05:00)

39. Approximately what time would you go to bed if you were entirely free to plan your evening?

8:00 PM–9:00 PM (20:00–21:00 h)	9:00 PM–10:15 PM (21:00–22:15 h)	10:15 PM–12:30 AM (22:15–00:30 h)	12:30 AM–1:45 AM (00:30–01:45 h)	1:45 AM–3:00 AM (01:45–03:00 h)	3:00 AM-8:00 PM (03:00-20:00 h)

40. If you usually have to get up at a specific time in the morning, how much do you depend on an alarm clock?

Not al all	Slightly	Somewhat	Very much

41. How easy do you find it to get up in the morning (when you are not awakened unexpectedly)?

Very difficult	Somewhat difficult	Fairly easy	Very easy

42. How alert do you feel during the first half hour after you wake up in the morning?

Not at all alert	Slightly alert	Fairly alert	Very alert

43. How hungry do you feel during the first half hour after you wake up?

Not at all hungry	Slightly hungry	Fairly hungry	Very hungry

44. During the first half hour after you wake up in the morning, how do you feel?

Very tired	Fairly tired	Fairly refreshed	Very refreshed

45. If you had no commitments the next day, what time would you go to bed compared to your usual bedtime?

Seldom or never later	Less than 1 hour later	1-2 hours later	More than 2 hours later

46. You have decided to do physical exercise. A friend suggests that you do this for one hour twice a week, and the best time for him is between 7-8 AM (07-08 h). Bearing in mind nothing but your own internal "clock," how do you think you would perform?

Would be in good form	Would be in reasonable form	Would find it difficult	Would find it very difficult

47. At approximately what time in the evening do you feel tired, and, as a result, in need of sleep?

8:00 PM–9:00 PM (20:00–21:00 h)	9:00 PM–10:15 PM (21:00–22:15 h)	10:15 PM–12:45 AM (22:15–00:45 h)	12:45 AM–2:00 AM (00:45–02:00 h)	2:00 AM–3:00 AM (02:00–03:00 h)

48. You want to be at your peak performance for a test that you know is going to be mentally exhausting and will last two hours. You are entirely free to plan your day. Considering only your "internal clock," which one of the four testing times would you choose?

8 AM–10 AM (08–10 h)	11 AM–1 PM (11–13 h)	3 PM–5 PM (15–17 h)	7 PM–9 PM (19–21 h)

49. If you got into bed at 11 PM (23 h), how tired would you be?

Not at all tired	A little tired	Fairly tired	Very tired

50. For some reason you have gone to bed several hours later than usual, but there is no need to get up at any particular time the next morning. Which one of the following are you most likely to do?

Will wake up at usual time, but will not fall back asleep	Will wake up at usual time and will doze thereafter	Will wake up at usual time, but will fall asleep again	Will not wake up until later than usual

51. One night you have to remain awake between 4-6 AM (04-06 h) in order to carry out a night watch. You have no time commitments the next day. Which one of the alternatives would suit you best?

- Would not go to bed until the watch is over
- Would take a nap before and sleep after
- Would take a good sleep before and nap after
- Would sleep only before the watch

52. You have two hours of hard physical work. You are entirely free to plan your day. Considering only your internal "clock," which of the following times would you choose?

- 8 AM–10 AM (08–10 h)
- 11 AM–1 PM (11–13 h)
- 3 PM–5 PM (15–17 h)
- 7 PM–9 PM (19–21 h)

53. You have decided to do physical exercise. A friend suggests that you do this for one hour twice a week. The best time for her is between 10-11 PM (22-23 h). Bearing in mind only your internal "clock," how well do you think you would perform?

- Would be in good form
- Would be in reasonable form
- Would find it difficult
- Would find it very difficult

54. Suppose you can choose your own work hours. Assume that you work a five-hour day (including breaks), your job is interesting, and you are paid based on your performance. At approximately what time would you choose to begin?

- 5 hours starting between 4–8 AM (05–08 h)
- 5 hours starting between 8–9 AM (08–09 h)
- 5 hours starting between 9 AM–2 PM (09–14 h)
- 5 hours starting between 2–5 PM (14–17 h)
- 5 hours starting between 5 PM–4 AM (17–04 h)

55. At approximately what time of day do you usually feel your best?

- 5–8 AM (05–08 h)
- 8–10 AM (08–10 h)
- 10 AM–5 PM (10–17 h)
- 5–10 PM (17–22 h)
- 10 PM–5 AM (22–05 h)

56. One hears about "morning types" and "evening types." Which one of these types do you consider yourself to be?

- Definitely a morning type
- Rather more a morning type than an evening type
- Rather more an evening type than a morning type
- Definitely an evening type

S4: PSQI questionnaire

The Pittsburgh Sleep Quality Index (PSQI)

Instructions: This questionnaire asks you about your sleep quality. Please attempt to answer every question. You do not have to answer any question you feel uncomfortable answering. Your answers should indicate the most accurate reply for the majority of days and nights in the past month.
During the past month,

63. When have you usually gone to bed?

	hh		mm	AM/PM
Time		:		-

64. How long (in minutes) has it taken you to fall asleep each night?

65. When have you usually gotten up in the morning?

	hh		mm	AM/PM
Time		:		-

66. How many hours of actual sleep do you get at night? (This may be different than the number of hours you spend in bed)

67. During the past month, how often have you had trouble sleeping because you…

	Not during the past month	Less than once a week	Once or twice a week	Three or more times a week
Cannot get to sleep within 30 minutes	○	○	○	○
Wake up in the middle of the night or early morning	○	○	○	○
Have to get up to use the bathroom	○	○	○	○
Cannot breathe comfortably	○	○	○	○
Cough or snore loudly	○	○	○	○
Feel too cold	○	○	○	○
Feel too hot	○	○	○	○
Have bad dreams	○	○	○	○
Have pain	○	○	○	○
Other reason(s)	○	○	○	○

68. During the past month, how often have you taken medicine (prescribed or "over the counter") to help you sleep?

Not during the past month	Less than once a week	Once or twice a week	Three or more times a week
○	○	○	○

69. During the past month, how often have you had trouble staying awake while driving, eating meals, or engaging in social activity?

Not during the past month	Less than once a week	Once or twice a week	Three or more times a week
○	○	○	○

70. During the past month, how much of a problem has it been for you to keep up enthusiasm to get things done?

Not during the past month	Less than once a week	Once or twice a week	Three or more times a week
○	○	○	○

71. During the past month, how would you rate your sleep quality overall?

Very good	Fairly good	Fairly bad	Very bad
○	○	○	○

S5: Results table of all correlation analyses

		Correlation analysis P-values				
Metric	Device	Fitbit	Hexoskin	SRSM	Withings	Oura
TSD	Fitbit	—	5.12e-13***	7.67e-09***	1.17e-12***	3.87e-07***
	Hexoskin	5.12e-13***	—	5.12e-09***	3.78e-12***	3.68e-04***
	SRSM	7.67e-09***	5.12e-09***	—	1.16e-08***	3.55e-03***
	Withings	1.17e-12***	3.78e-12***	1.16e-08***	—	7.94e-07***
	Oura	3.87e-07***	3.68e-04***	3.55e-03***	7.94e-07***	—
REM	Hexoskin	—	—	—	1.98e-04***	1.24e-02**
	Withings	—	1.98e-04***	—	—	1.06e-06***
	Oura	—	1.24e-02**	—	1.06e-06***	—
Overall Sleep Stages	Hexoskin	—	—	—	<2.20e-16***	<2.20e-16***
	Withings	—	<2.20e-16***	—	—	<2.20e-16***
	Oura	—	<2.20e-16***	—	<2.20e-16***	—

Note: * P < 0.1; ** P < 0.05; *** P < 0.01; TSD refers to total sleep duration; REM refers to rapid eye movement sleep stage; SRSM refers to self-reported sleep metrics. Note that the results from the Kendall’s rank correlation test returned P values below the threshold of reporting in the R programming language and thus were reported as less than the threshold returned in the test outputs.

S6: Univariate analyses comparing morning cognitive scores to device data

		Morning Cognitive Scores			
Device	Feature	Coefficient	Std. Error	P-Value	R^2
Fitbit	TSD	-0.003	0.014	0.825	0.004
	Wakeups	-0.018	0.018	0.329	0.068
Withings	TSD	-0.018	0.010	0.110	0.172
	Latency	.015	0.091	0.869	0.002
	Efficiency	-0.001	0.001	0.315	0.072
	Wakeups	.001	0.001	0.888	0.001

	REM	-0.039	0.040	0.342	0.065
Oura	TSD	-0.022	0.019	0.265	0.088
	Latency	-0.105	0.113	0.366	0.059
	Efficiency	0.002	0.002	0.285	0.081
	Wakeups	-0.018	0.014	0.226	0.103
	REM	-0.008	0.0173	0.656	0.015
Hexoskin	TSD	-0.020	0.015	0.206	0.112
	Latency	-0.001	0.096	0.995	0.000
	Efficiency	0.003	0.005	0.530	0.029
	REM	-0.071	0.044	0.128	0.158
SRSM	TSD	-0.005	0.014	0.725	0.009
	Latency	-0.008	0.091	0.935	0.000
Observations		16			

Note: * P < 0.1; ** P < 0.05; *** P < 0.01; SRSM refers to self-reported sleep metrics.

S7: Univariate analyses comparing afternoon cognitive scores to device data

		**Afternoon Cognitive Scores**			
Device	Feature	Coefficient	Std. Error	P-Value	R^2
Fitbit	TSD	-0.016	0.024	0.511	0.026
	Wakeups	0.014	0.033	0.672	0.011
Withings	TSD	-0.013	0.019	0.497	0.028
	Latency	-0.272	0.101	0.0158**	0.297
	Efficiency	0.003	0.002	0.148	0.099
	Wakeups	-0.0153	0.0163	0.361	0.049
	REM	-0.036	0.071	0.617	0.015
Oura	TSD	0.036	0.027	0.197	0.096
	Latency	0.138	0.200	0.499	0.027
	Efficiency	0.004	0.004	0.332	0.055
	Wakeups	0.025	0.018	0.184	0.101
	REM	0.010	0.028	0.732	0.007
Hexoskin	TSD	-0.028	0.026	0.289	0.066
	Latency	0.113	0.157	0.481	0.030
	Efficiency	-0.006	0.009	0.527	0.024
	REM	-0.103	0.074	0.186	0.101
SRSM	TSD	-0.022	0.023	0.361	0.049
	Latency	0.197	0.151	0.210	0.091
Observations		19			

Note: * P < 0.1; ** P < 0.05; *** P < 0.01; SRSM refers to self-reported sleep metrics.

S8: Univariate analyses comparing evening cognitive scores to device data

		Evening Cognitive Scores			
Device	Feature	Coefficient	Std. Error	P-Value	R^2
Fitbit	TSD	-0.012	0.023	0.61	0.017
	Wakeups	0.006	0.032	0.857	0.002
Withings	TSD	-0.016	0.018	0.409	0.043
	Latency	-0.271	0.097	0.013**	0.327
	Efficiency	0.002	0.002	0.194	0.103
	Wakeups	-0.015	0.016	0.378	0.049
	REM	-0.041	0.069	0.557	0.022
Oura	TSD	0.034	0.026	0.221	0.092
	Latency	0.116	0.196	0.563	0.021
	Efficiency	0.004	0.004	0.301	0.067
	Wakeups	0.021	0.019	0.289	0.070
	REM	0.011	0.028	0.713	0.009
Hexoskin	TSD	-0.031	0.025	0.235	0.087
	Latency	0.057	0.156	0.718	0.008
	Efficiency	-0.002	0.009	0.798	0.004
	REM	-0.108	0.077	0.180	0.109
SRSM	TSD	-0.025	0.022	0.273	0.075
	Latency	0.177	0.152	0.261	0.078
Observations		18			

Note: * P < 0.1; ** P < 0.05; *** P < 0.01; SRSM refers to self-reported sleep metrics.

S9: Univariate analyses comparing morning cognitive scores to participant summary data

	Morning Cognitive Scores			
Feature	Coefficient	Std. Error	P-Value	R^2
PSQI	-0.003	0.005	0.531	0.029
MEQ	-0.001	0.002	0.529	0.029
Emotional Role Limitations	0.0003	0.001	0.665	0.014
Energy	0.0003	0.001	0.700	0.011
General Health	-0.001	0.004	0.769	0.006
Physical	0.003	0.001	0.014**	0.358
Social	0.002	0.001	0.170	0.130

Well-being	0.002	0.001	0.078*	0.206
Observations	16			

Note: * P < 0.1; ** P < 0.05; *** P < 0.01.

S10: Univariate analyses comparing afternoon cognitive scores to participant summary data

	**Afternoon Cognitive Score**			
Feature	Coefficient	Std. Error	P-Value	R^2
PSQI	-0.013	0.007	0.083*	0.167
MEQ	0.005	0.003	0.057*	0.197
Emotional Role Limitations	0.002	0.001	0.005***	0.382
Energy	0.003	0.001	0.018**	0.288
General Health	-0.002	0.007	0.823	0.003
Physical	0.001	0.330	0.745	0.006
Social	0.005	0.001	0.004***	0.397
Well-being	0.004	0.001	0.005***	0.379
Observations	19			

Note: * P < 0.1; ** P < 0.05; *** P < 0.01.

S11: Univariate analyses comparing evening cognitive scores to participant summary data

	**Evening Cognitive Scores**			
Feature	Coefficient	Std. Error	P-Value	R^2
PSQI	-0.014	0.007	0.057**	0.208
MEQ	0.004	0.003	0.12	0.144
Emotional Role Limitations	0.002	0.001	0.003***	0.438
Energy	0.003	0.001	0.010***	0.345
General Health	0.0003	0.008	0.961	0.0001
Physical	0.001	0.003	0.597	0.018
Social	0.005	0.001	0.002***	0.463
Well-being	0.004	0.001	0.001***	0.500
Observations	18			

Note: * P < 0.1; ** P < 0.05; *** P < 0.01.
